# Peritoneal and renal DKK3 clearance in peritoneal dialysis

**DOI:** 10.1186/s12882-024-03715-7

**Published:** 2024-08-23

**Authors:** Hagen Ehleiter, Julia Miranda, Dominik Boes, Uta Scheidt, Sibylle von Vietinghoff, Sebastian Schwab

**Affiliations:** 1 Nephrology Section, Medical Clinic 1, University Hospital Bonn, Rheinische Friedrich- Wilhelms Universität Bonn, Venusberg Campus 1, D-53127 Bonn, Germany; 2 Kuratorium for Dialysis, KfH Renal Center, Bonn, Germany

**Keywords:** Peritoneal dialysis, DKK3, RAAS blockade

## Abstract

**Background:**

Urinary Dickkopf 3 (DKK3) excretion is a recently established biomarker of renal functional development. Its excretion into the peritoneal cavity has not been reported. We here studied DKK3 in peritoneal dialysis.

**Methods:**

DKK3 was assessed in serum, urine and dialysate in a prevalent adult peritoneal dialysis cohort and its concentration analyzed in relation to creatinine and clinical characteristics.

**Results:**

Highest DKK3 concentrations were found in serum, followed by urine. Dialysate concentrations were significantly lower. Dialysate DKK3 correlated with both other compartments. Serum, dialysate and urine values were stable during three months of follow-up. Continuous ambulatory dialysis (CAPD) but not cycler-assisted peritoneal dialysis (CCPD) volume-dependently increased peritoneal DKK3 in relation to creatinine. RAAS blockade significantly decreased urinary, but not serum or peritoneal DKK3.

**Conclusion:**

Our data provide a detailed characterization of DKK3 in peritoneal dialysis. They support the notion that the RAAS system is essential for renal DKK3 handling.

## Introduction

Peritoneal dialysis is a major mode of renal replacement worldwide [1]. Patient autonomy, lesser amounts of nursing workforce and independence of local water supply contribute to recent reappreciation in diverse world regions [2]. However, the use of the peritoneal membrane as biological dialysis filter also confers limitations. Functional deterioration with time on dialysis is observed in most patients [3]. Clearance assessment of different uremic and other solutes is also challenging for a dynamic biological membrane [4]. Diverse dialysis solutions, filling volumes and dwell times further enhance this complexity.

Most patients require at least some residual kidney function to reach an adequate peritoneal dialysis dose. Prediction of velocity of further GFR loss may therefore aid in advising on dialysis modality. Multiple biomarkers are currently studied for CKD and after acute kidney injury, mostly before dialysis commencement [5–7]. Among them, Dickkopf-3 (DKK3), a member of the WNT-beta catenin signaling pathway [8], was primarily investigated as a marker and possible pathophysiologic mediator of heart failure [9, 10], albeit with partially conflicting results. More recently, a DKK3 role in regeneration of various mesodermal tissues was determined [11]. DKK3 is an established promotor of kidney fibrosis [12] and a well appreciated marker for progression of renal disease [13]. Indeed, DKK3 mechanistically promoted renal fibrosis development in an animal model [14].

In humans, higher urinary DKK3/creatinine ratios associated with renal functional decline in CKD [15] and acute kidney injury after cardiac surgery [16]. In CKD outpatients, levels increased with more severe reduction of kidney function in cross-sectional study [17]. DKK3 is mainly secreted by tubular cells in the kidney. Consistently, urinary DKK3 levels also strongly correlated with cyst development in autosomal dominant polycystic kidney disease [18]. A correlation of urinary DKK3 with GFR loss also applies to children with CKD of diverse etiologies [19]. The latter study introduced the notion that urinary DKK3 is suppressed significantly by RAAS blockade.

Less is known in patients with CKDG5. In kidney transplant recipients, DKK3 was successful in predicting future functional decline in one study [20], but not superior to GFR in predicting outcome in another investigation [21]. In patients receiving peritoneal dialysis, one report [22] found a negative association of urinary DKK3 with subsequent renal functional decline with a large variability of individual values.

DKK3 levels in the peritoneal dialysate, their relation to systemic and urinary levels, and to peritoneal dialysis regimens have not been described. We therefore assessed serum, urinary and dialysate DKK3 concentrations in a cohort of adult peritoneal dialysis patients.

## Methods

### Patient recruitment and treatment description

Prevalent adult peritoneal dialysis outpatients without active infection were recruited after ethics board approval and informed consent at our local center (376/21). Clinical information including standard laboratory values were extracted from the records. Dialysis efficacy was estimated using weekly Kt/V ratio according to the National Cooperative Dialysis Study (NCDS) [23].

### DKK3 assessment

Fresh serum, 24 h urine and dialysate samples were centrifuged at 2,000 xg for 10 min and the supernatant was kept at -80 °C until analysis using Human Dkk-3 DuoSet ELISA Kit (Cat #DY1118, R&D Systems) originally designed for cell culture supernatant, serum and plasma according to manufacturer’s instructions.

### Statistical analysis

Two-tailed student’s t-test was used to compare two conditions, Welch’s correction for unequal variance or Mann-Whitney for non-parametric values were employed as indicated. If more than two conditions were compared, Dunnet’s test after ANOVA or non-parametric test was employed as appropriate and indicated in the figure legends. These and correlation and regression analyses were calculated using GraphPadPrism (Irvine, CA). Data are expressed as mean ± SEM. P-values < 0.05 were considered significant and are indicated: *p < 0.05, **p < 0.01, ***p < 0.001.

## Results

### DKK3 is cleared poorly in peritoneal dialysis fluid

**Fig. 1:**
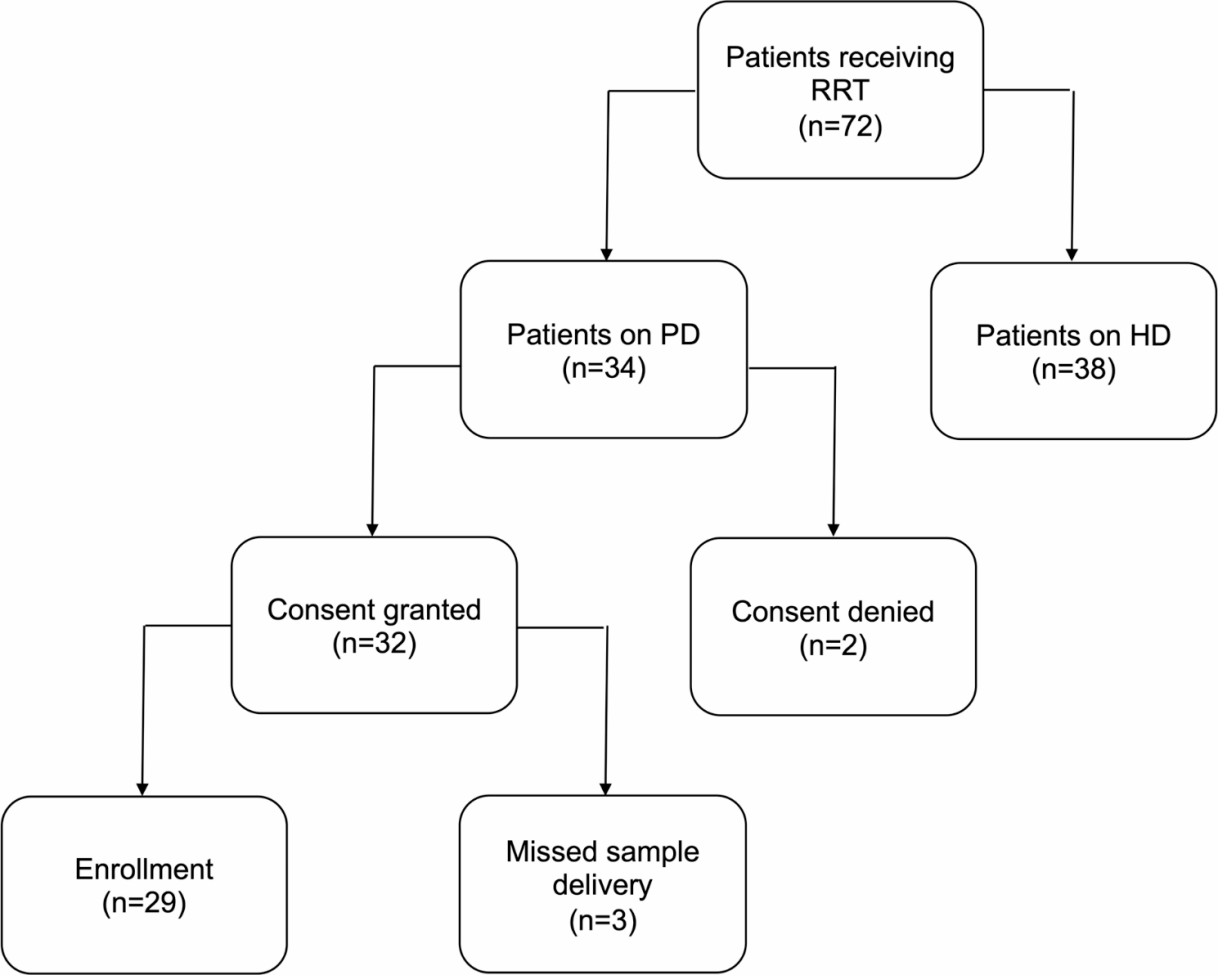
Flow diagram of patient recruitment. *RRT* renal replacement therapy, *PD* peritoneal dialysis, *HD* hemodialysis

DKK3 concentrations in serum, urine and peritoneal dialysate were assessed in a cohort of prevalent peritoneal dialysis outpatients (Fig. 1). We succeeded in recruiting 85% of patients at our center. Mean age was 56 years and 52% of the patients were female (Table 1). Most common causes of kidney disease were hypertension and glomerulonephritis, while diabetic nephropathy was not among diagnoses. Further standard laboratory values are shown in Table 2. Mean peritoneal dialysis vintage was 1354 days, with similar proportions receiving continuous ambulatory dialysis (CAPD) and cycler-assisted peritoneal dialysis (CCPD) regimens (Table 2).

**Table 1:** Clinical characteristics (*n* = 29)

	% (*n*) or mean ± SEM
**Basic characteristics**	
Age	55.8 ± 3.3 years
Gender	48% (14) female
Dialysis vintage	1354 ± 265 days
BMI	24.4 ± 0.7
BSA (DuBois, m^2^)	1.86 ± 0.03
**Underlying renal disease**	
Diabetic nephropathy	0% (0)
Hypertensive nephropathy	21% (6)
Glomerulonephritis	27% (8)
Miscellaneous (cystic kidney diseases, reflux etc.)	34% (10)
Unknown	17% (5)
**Comorbidities**	
Diabetes mellitus	7.0% (2)
Hypertension	44.8% (13)
Coronary artery disease	14.0% (4)
Cerebrovascular or peripheral artery disease	21.0% (6)
Malignoma	7.0% (2)
**Medication**	
Erythropoiesis stimulating agent	58.6% (17)
RAAS blockade (ACEi, ARB or MRA)	76.9% (22)
Diuretic (without MRA)	65.5% (19)

*ACEi* ACE inhibitor, *ARB* Angiotensin receptor blocker, *MRA* aldosterone antagonist

**Table 2:** Laboratory and dialysis characteristics of the patient cohort (*n* = 29)

	% (*n*) or mean ± SEM
**Laboratory values**	
Hemoglobin	10.3 ± 0.2 g/dL
Leukocytes	7.5 ± 0.4*10^3^/µl
CRP	5.1 ± 1.3 mg/dL
Albumin	35.2 ± 1.0 g/L
Creatinine	9.2 ± 0.7 mg/dL
Phosphorous	1.8 ± 0.1 mmol/L
Uric acid	6.0 ± 0.2 mg/dL
Proteinuria (rel. to creatinine)	0.92 ± 0.2 g/g
**Dialysis**	
CAPD/CCPD/IPD	48% (14)/45%(13)/7%(2)
Residual urine output (% of pts)	79% (23)
Mean urine volume (if present)	1268 ± 138 mL/d
Dialysate volume	10.5 ± 0.5 L/24 h
Weekly dialysis clearance (Watson’s Kt/V)	2.21 ± 0.2
Renal creatinine clearance	3.9 ± 0.6 mL/min
Peritoneal creatinine clearance	34.4 ± 17.9 L/week/m^2^

**Fig. 2:**
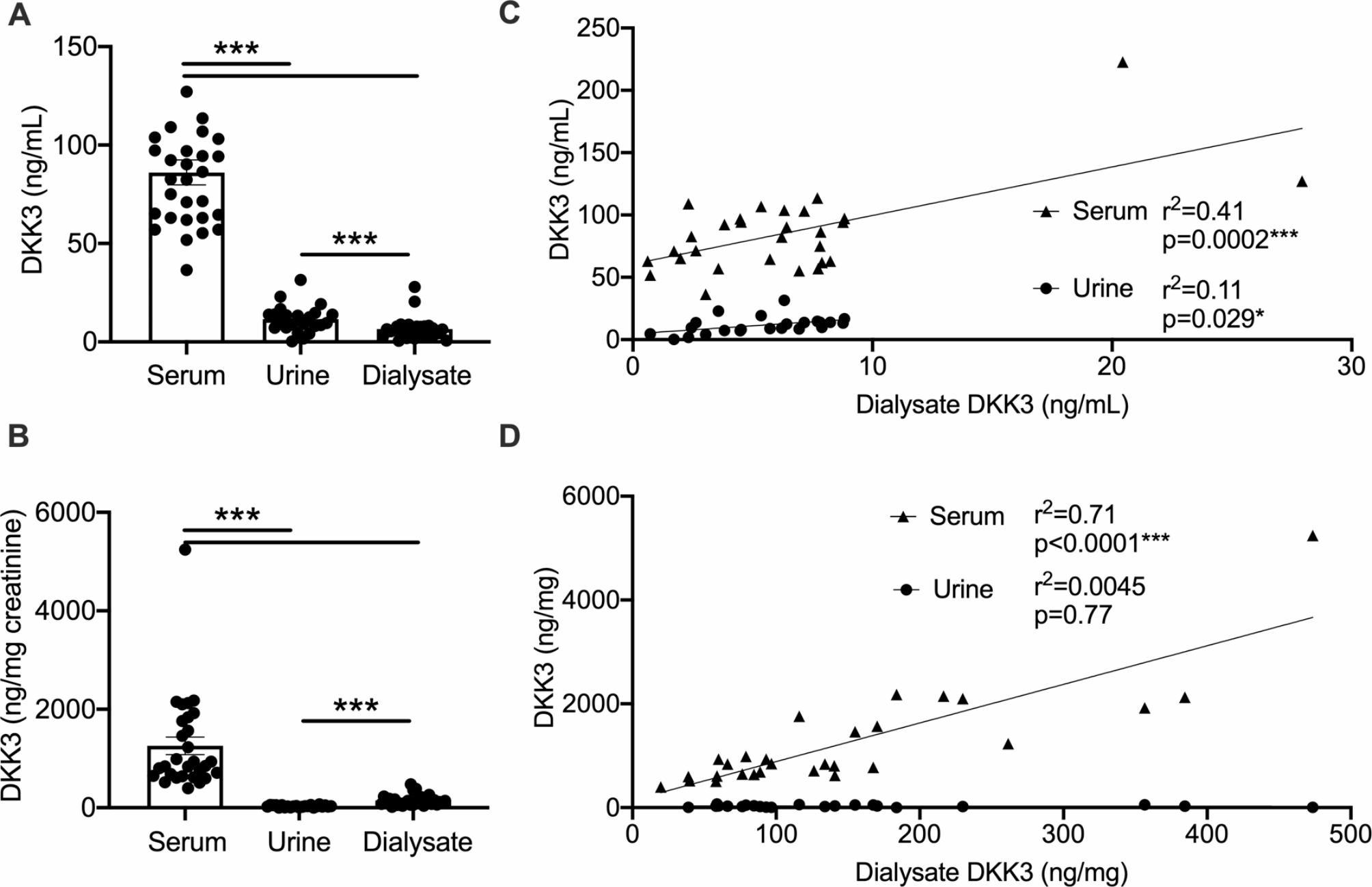
Peritoneal dialysate DKK3 correlates with serum and urine. **(A**-**D)** DKK3 levels were assessed in serum, urine and peritoneal dialysate as absolute concentrations and relative to creatinine. (A, B) Absolute (A) and concentrations relative to creatinine (B) (*n* = 29 patients, Tukey’s after ANOVA). (C, D) Correlations of absolute (C) and relative to creatinine (D) dialysate concentrations to serum and urine (results of linear regression analyses are shown)

DKK3 concentrations were highest in serum (Fig. 2A). Urine absolute concentrations were higher than in peritoneal dialysate. Urinary DKK3/creatinine ratio is the most common measure employed in current literature [9]. It was significantly lower in urine than peritoneal dialysate, driven by urine concentration of creatinine (Fig. 2B). Serum DKK3/creatinine ratio was by far higher than in the two other compartments, despite the expectedly elevated serum creatinine in peritoneal dialysis (Table 2). Dialysate DKK3 levels correlated with both urine and serum (Fig. 2C), while DKK3 in relation to creatinine in dialysate was similar to serum, but not urine (Fig. 2D).

These results establish DKK3 peritoneal dialysate concentrations in a cohort of stable adult outpatients.

### DKK3 concentrations are longitudinally stable in end stage kidney disease managed with peritoneal dialysis

**Fig. 3:**
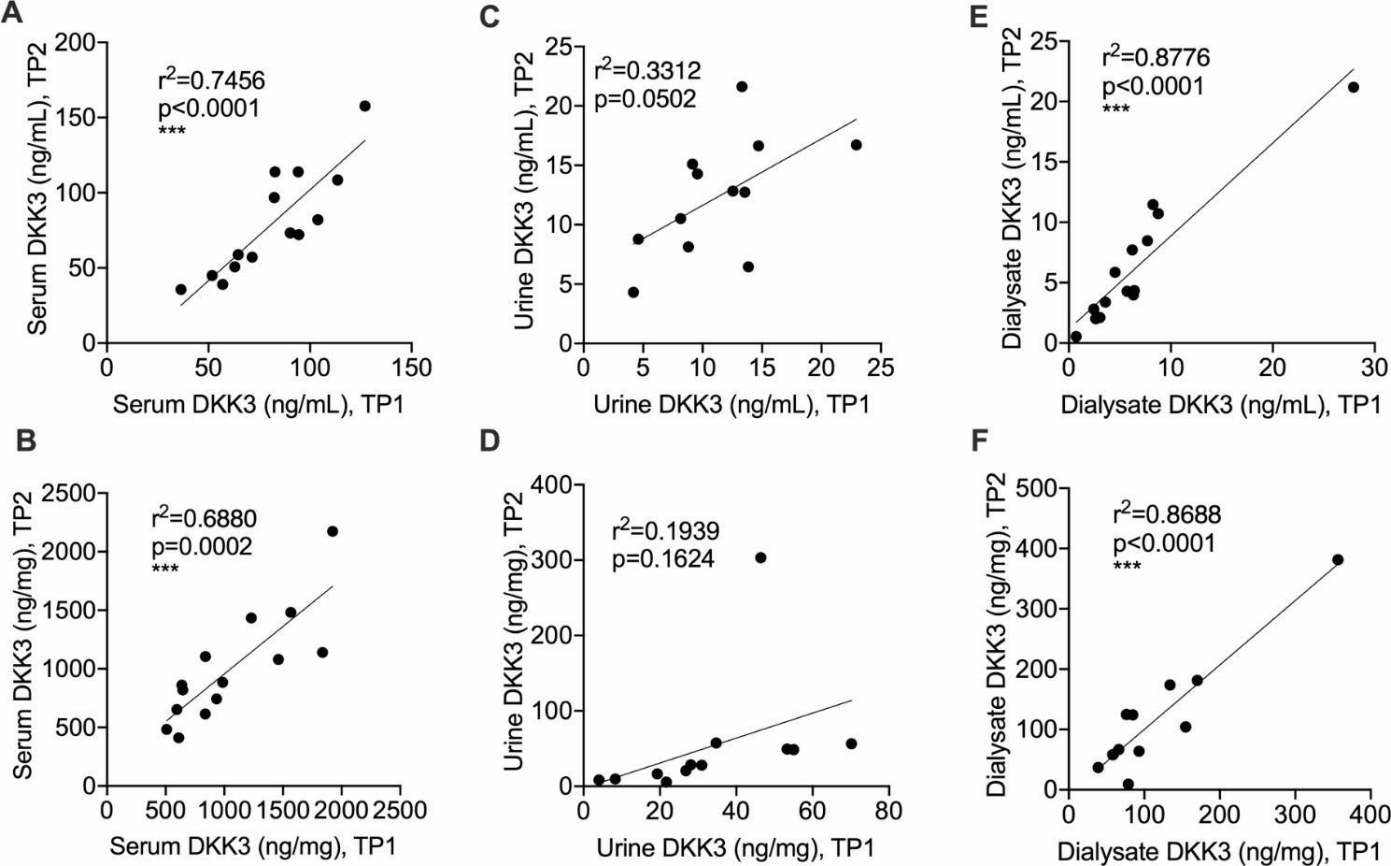
DKK3 concentrations are stable in peritoneal dialysis outpatients. (**A**-**F**) DKK3 levels were assessed in serum, urine and peritoneal dialysate as absolute concentrations and relative to creatinine. Correlations of longitudinal DKK3 assessments at least three months apart in serum (A, B), urine (C, D) and peritoneal dialysate (E, F) are given as absolute concentrations (A, C, E) and relative to creatinine (B, D, F) (results of linear regression analyses are shown). *TP* time point

DKK3 measurements were longitudinally repeated in a subgroup of 14 patients. During three months, values did not change significantly in either compartment. Sequentially assessed DKK3 levels in serum and peritoneal dialysate significantly linearly correlated (Fig. 3A-E).

We also tested whether dialysate DKK3 concentration was related to time on peritoneal dialysis. There was no significant association with dialysis vintage (Pearson’s r for ng DKK3/mL = 0.25, *p* = 0.22 and for ng DKK3/mg creatinine=-0.046, *p* = 0.82). However, DKK3 dialysate concentration changes were larger in individual patients that were studied in early peritoneal dialysis (Pearson’s r for DKK3 (mg/mL) and dialysis vintage = 0.68, *p* = 0.007). Dialysate DKK3 in relation to creatinine was unchanged.

Overall, DKK3 levels were stable in all investigated compartments in this peritoneal dialysis cohort.

### Dialysate DKK3 concentrations in CAPD correlate with dialysate volume per day

**Fig. 4:**
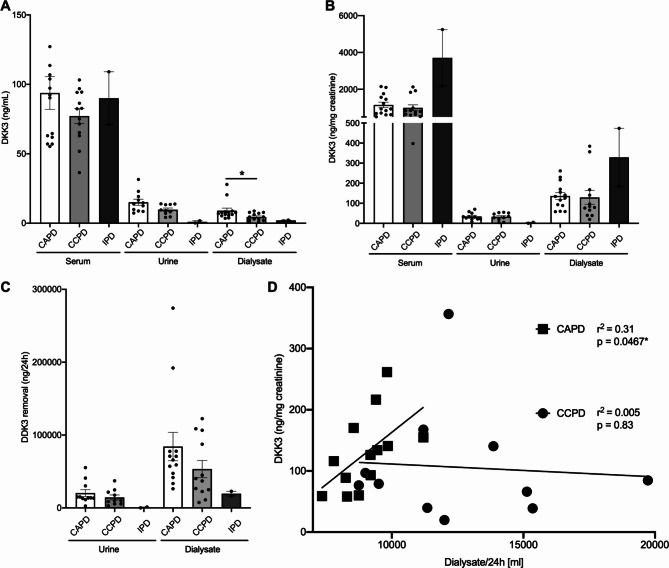
Peritoneal DKK3 clearance depending on dialysis regimen. **(A**,** B)** Peritoneal dialysate DKK3 absolute (A) and relative to creatinine (B) levels according to CAPD, CCPD and IPD regimens (statistical analysis of CAPD versus CCPD: t-test with Welch’s correction). (C) DKK3 urine and peritoneal removal in mg/24 h according to dialysis regimen. (D) Dialysate DKK3 levels in for CAPD and CCPD in relation to dialysate volume (linear regression analyses)

We next investigated if peritoneal DKK3 clearance associates with peritoneal dialysis modality. Patients receiving CAPD were compared to CCPD. Our cohort also includes two individuals on intermittent PD (IPD), whose results are also shown in Fig. 4.

Serum and urine DKK3 concentrations were very similar for CAPD and CCPD (Fig. 4A). However, peritoneal dialysate DKK3 levels were significantly higher in CAPD. The relation of DKK3 and creatinine did not differ in dialysate, urine or serum of the two groups (Fig. 4B). Dialysate levels were also very similar in patients classified as low average, high average and high transporters (data not shown).

We tested for differences in total daily DKK3 removal via urine and dialysate in CAPD and CCPD groups. No significant differences were found (Fig. 4C). However, daily dialysate volumes were significantly larger for CCPD than CAPD (12 ± 0.9 L versus 9 ± 0.3 L, *p* = 0.0045, Mann-Whitney test), as expected. If these groups were investigated separately, daily DKK3 removal in relation to creatinine significantly correlated with volume in CAPD, but not CCPD (Fig. 4D).

These data demonstrate differential DKK3 and creatinine peritoneal recovery in patients treated with larger volumes via CAPD.

### Decreased urinary DKK3 during RAAS blockade in peritoneal dialysis patients

**Fig. 5:**
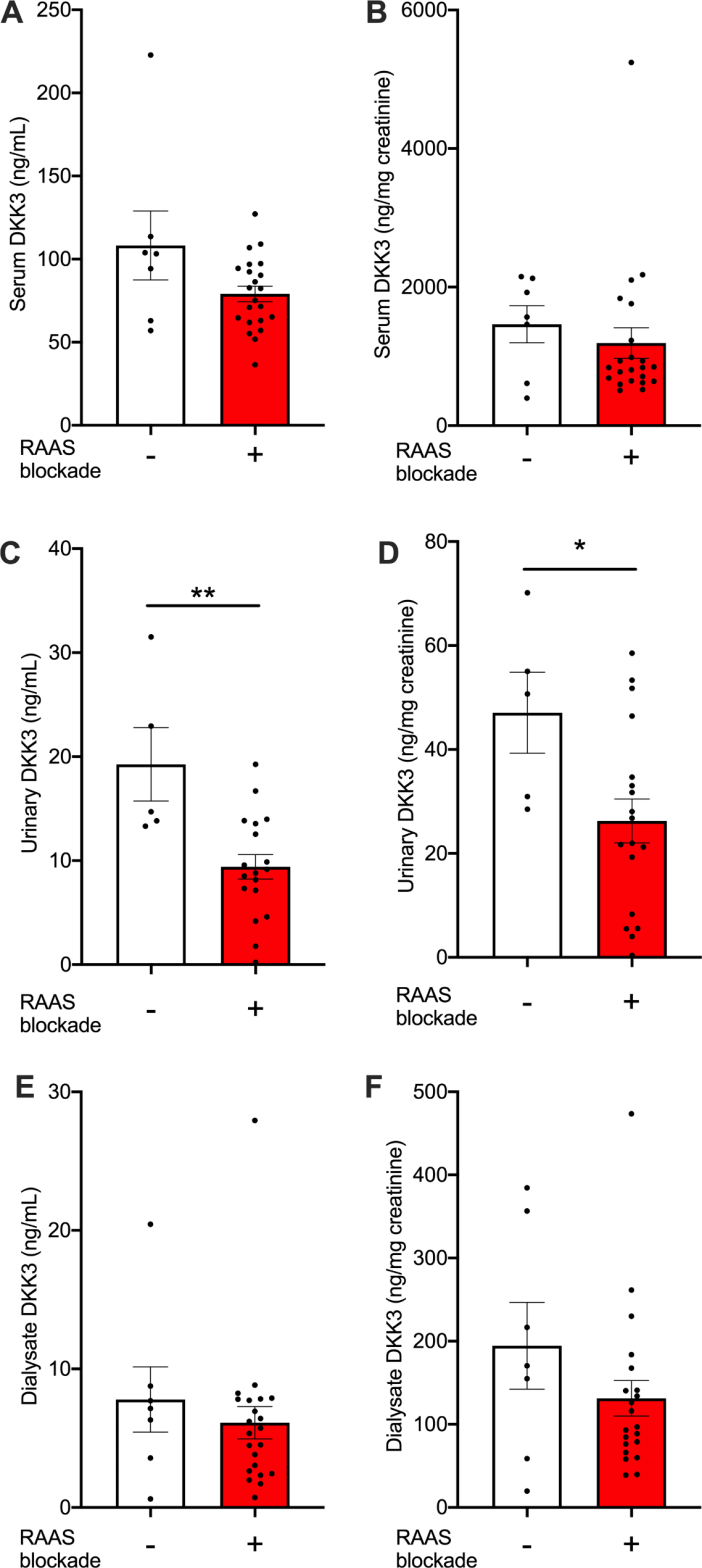
Decreased urinary DKK3 excretion during RAAS blockade. **(A**-**E)** DKK3 levels in serum (A, B), urine (C, D) and peritoneal dialysate (E, F) in relation to RAAS blockade are shown as absolute concentrations (A, C, E) and relative to creatinine (B, D, F)(*n* = 7 patients without and *n* = 22 with blockade (*n* = 18 ACEi or ARB, *n* = 4 *MRA* Mann-Whitney tests)

To test whether urinary DDK3 excretion is modulated by RAAS blockade also in adult patients with dialysis-dependent renal disease [19], we determined RAAS blockade in our cohort. 76% received either an ACE inhibitor (ACEi) or angiotensin receptor blocker (ARB, *n* = 18), or aldosterone antagonist (MRA, *n* = 4). Their DKK3 serum levels did not differ from patients without RAAS blockade (Fig. 5A, B). However, urine levels, both absolute concentrations and in relation to creatinine, were significantly lower (Fig. 5C, D). Very similar trends were seen for ACEi, ARB and MRA (data not shown). RAAS blockade did not detectably impact on peritoneal DKK3 removal (Fig. 5E, F).

These data extend a recent observation on urinary DKK3 excretion in children with CKD to a dialysis population.

## Discussion

Our data provide a first detailed description of DKK3 peritoneal and urinary excretion in peritoneal dialysis.

DKK3 dialysate concentrations were stable in all studied compartments. Dialysate DKK excretion was moderate. Urinary excretion levels were similar to recently reported pediatric CKD cohort without dialysis [19]. Our data also confirm the recent negative association of urinary DKK3 excretion with RAAS blockade. Interestingly, the amount of DKK3 reduction in our adult peritoneal dialysis cohort was in the same order of magnitude as reported for children without renal replacement. RAAS blockade is beneficial for preservation of residual kidney function in peritoneal dialysis [24]. It is conceivable that reduced urinary DKK3 levels reflect diminished renal stress responses also in peritoneal dialysis, however, mechanistic data remain to be obtained. Whether urinary DKK3 predicts preservation of residual kidney function in manners additional to GFR [21] and proteinuria in peritoneal dialysis now needs to be studied prospectively regarding both, urine volume and solute clearance.

DKK3 is produced and secreted in multiple organs, mostly by fibroblasts, but also other cell types including renal tubular epithelium [25]. It was investigated most closely as a fibrotic and cell stress marker in heart and kidney [9]. To the best of our knowledge, our report is first to present data on the peritoneal cavity in peritoneal dialysis. RAAS inhibition with an otherwise beneficial impact on the peritoneal function in dialysis [26] did not affect peritoneal DKK3. We found higher levels in CAPD than CCPD, which may be related to longer dwell times. In addition, peritoneal DKK3 associated with total volumes in CAPD. This is somewhat reminiscent of cardiac DKK3 expression by the strained cardiac tissue in heart failure [10]. Peritoneal mesothelial DKK3 response to strain should be investigated.

Taken together, peritoneal DKK3 removal by dialysis is minor. Our data suggest that DKK3 in urine remains responsive to RAAS blockade also in end stage kidney disease treated with peritoneal dialysis. Increased peritoneal DKK3 accumulation in CAPD warrants further investigation as a peritoneal stress and possibly fibrosis indicator.

## Data Availability

All data generated or analyzed during this study are included in this published article.
